# Non-destructive, label free identification of cell cycle phase in cancer cells by multispectral microscopy of autofluorescence

**DOI:** 10.1186/s12885-019-6463-x

**Published:** 2019-12-21

**Authors:** Jared M. Campbell, Abbas Habibalahi, Saabah Mahbub, Martin Gosnell, Ayad G. Anwer, Sharon Paton, Stan Gronthos, Ewa Goldys

**Affiliations:** 1 Department of Physics and Astronomy, Macquarie University, North Ryde, New South Wales 2109 Australia; 2 ARC Centre of Excellence for Nanoscale BioPhotonics, Macquarie University, North Ryde, New South Wales 2109 Australia; 3 ARC Centre of Excellence in Nanoscale Biophotonics, The University of New South Wales, Sydney, New South Wales 2052 Australia; 4 Graduate School of Biomedical Engineering, The University of New South Wales, Sydney, New South Wales 2052 Australia; 5 School of Engineering, Faculty of Science and Engineering, Macquarie University, 2109, North Ryde, NSW 2109 Australia; 6 Quantitative Pty Ltd, Mt Victoria, New South Wales 2786 Australia; 7 Mesenchymal Stem Cell Laboratory, Adelaide Medical School, Faculty of Health and Medical Sciences, University of Adelaide, Adelaide, South Australia 5000 Australia; 8 South Australian Health and Medical Research Institute, Adelaide, South Australia 5000 Australia

**Keywords:** Cell phase, Cell cycle, Multispectral, Hyperspectral, Neoplasia, Cancer

## Abstract

**Background:**

Cell cycle analysis is important for cancer research. However, available methodologies have drawbacks including limited categorisation and reliance on fixation, staining or transformation. Multispectral analysis of endogenous cell autofluorescence has been shown to be sensitive to changes in cell status and could be applied to the discrimination of cell cycle without these steps.

**Methods:**

Cells from the MIA-PaCa-2, PANC-1, and HeLa cell lines were plated on gridded dishes and imaged using a multispectral fluorescence microscope. They were then stained for proliferating cell nuclear antigen (PCNA) and DNA intensity as a reference standard for their cell cycle position (G1, S, G2, M). The multispectral data was split into training and testing datasets and models were generated to discriminate between G1, S, and G2 + M phase cells. A standard decision tree classification approach was taken, and a two-step system was generated for each line.

**Results:**

Across cancer cell lines accuracy ranged from 68.3% (MIA-PaCa-2) to 73.3% (HeLa) for distinguishing G1 from S and G2 + M, and 69.0% (MIA-PaCa-2) to 78.0% (PANC1) for distinguishing S from G2 + M. Unmixing the multispectral data showed that the autofluorophores NADH, FAD, and PPIX had significant differences between phases. Similarly, the redox ratio and the ratio of protein bound to free NADH were significantly affected.

**Conclusions:**

These results demonstrate that multispectral microscopy could be used for the non-destructive, label free discrimination of cell cycle phase in cancer cells. They provide novel information on the mechanisms of cell-cycle progression and control, and have practical implications for oncology research.

## Background

Dividing cells must pass through the four phases of the cell cycle to duplicate their DNA and separate into two daughter cells. These phases are gap 1 (G1) during which the cell grows, increasing protein content and organelles; synthesis (S) during which nuclear DNA is replicated; gap 2 (G2) a second growth phase; and then mitosis (M) where cell division occurs. Progression through the phases is controlled by checkpoints, most notably at the G1-S and G2-M transitions [1].

Cell-cycle phase identification is important for the basic investigation of the growth characteristics of cell lines, especially in cancer research where the cellular mechanisms of cell growth and division may offer therapeutic opportunities [1]. Broadly, oncotherapies target dividing cells while sparing non-dividing cells (consequently achieving a degree of neoplastic specificity). However, some therapies are sensitive to cell cycle phase, such as methotrexate which induces S-phase arrest [2] or radiation therapy, most effective when cells are at the G2-M transition and least effective during the latter stages of S-phase [3]. Consequently, the assessment of cell cycle distribution in tumours may help enable personalised therapy by informing the selection of therapeutic strategies which they are optimally vulnerable to.

**Fig. 1:**
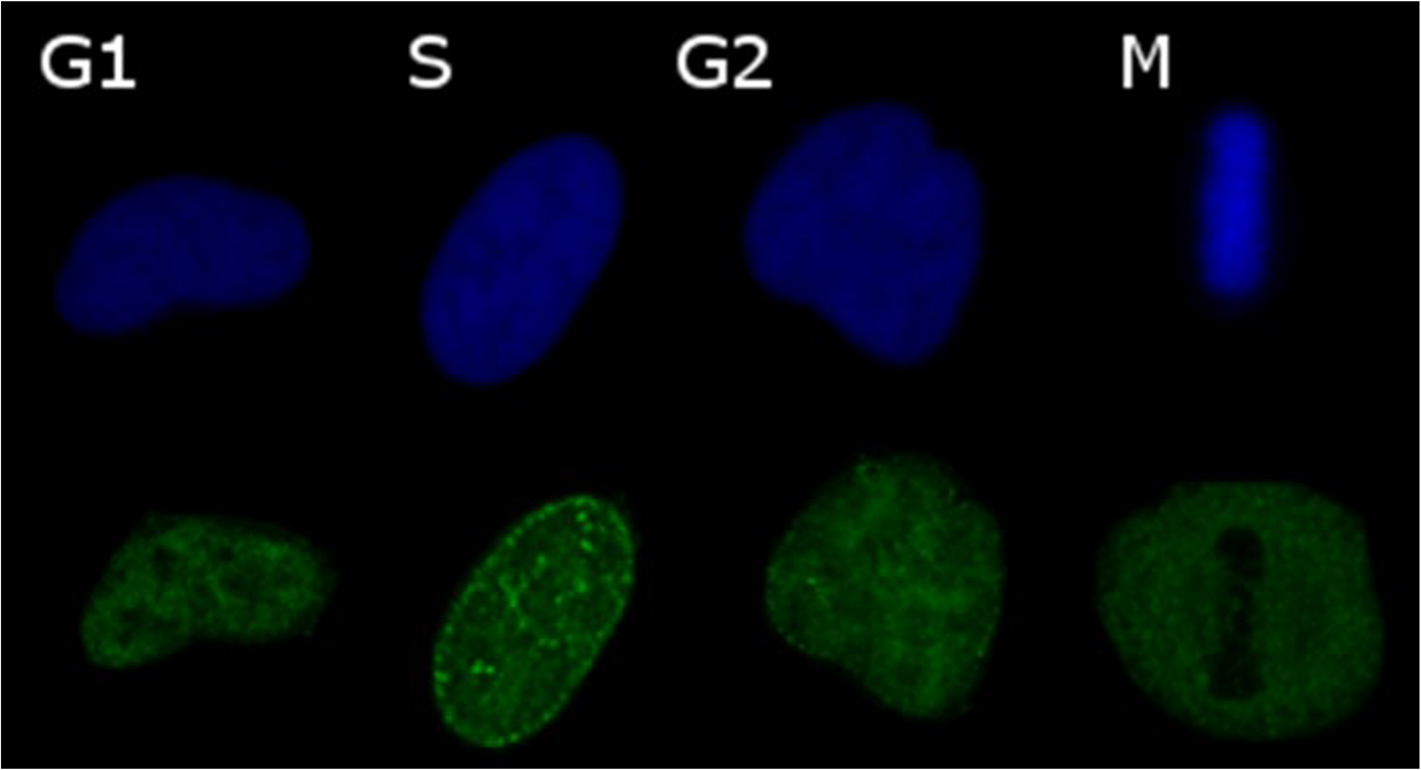
Confocal laser scanning images of phases of the cell cycle. HeLa nuclei, blue is DAPI green is PCNA. G1 phase is distinguished by solid distribution of PCNA through the nucleus, S phase is distinguished by PCNA speckling through the nucleus and a nuclear border depending on position within S-phase (mid-S displayed here) as well as increased total DNA intensity compared to G1, G2 is distinguished by solid distribution of PCNA and twice the total DNA intensity of G1, M phase is distinguished by the exclusion of PCNA from the nucleus into the cytoplasm

A routine methodology for investigating the cell cycle is staining cells with a DNA specific fluorescent probe (i.e. DAPI or Hoechst), with or without fixation. Fluorescence intensity then shows whether cells are pre, post or in the process of DNA replication allowing G1, S and G2/M phases to be determined. Flow cytometry is then typically used to assess distribution between the phases at a population level, while microscopy can be applied for the identification of individual cells [4]. Finer, more definitive categorisation can be achieved using markers of cell cycle phase such as proliferating cell nuclear antigen (PCNA), an essential component for DNA replication [5], whose distribution pattern changes with the phases of the cell cycle (Fig. 1) and, in combination with measurement of DNA fluorescence intensity, distinguishes G1, S, G2 and M-phase cells [6]. In combination with Ki-67 PCNA can also be used for the assessment of cell-cycle in flow cytometry [7]. The fluorescence ubiquitination cell cycle indicator (FUCCI) system uses reporter genes that encode fluorescing proteins that indicate G1, G1 to S transition and S/G2/M [8]. All of these systems have drawbacks, however, including limited ability to distinguish certain phases, removal from culture, stain toxicity, fixation, and transformation. Additionally, any reporter fluorophore used to indicate cell cycle phase reduces the number of potential labels that can be simultaneously used on a fluorescent microscope, which potentially limits investigations.

Cells contain numerous endogenous fluorophores that can be directly distinguished by their unique excitation and emission profiles without use of exogenous labels or indicators [9–11]. Some of these autofluorophores can provide useful information on intracellular activity. For example, the autofluorescent coenzymes nicotinamide adenine dinucleotide (NAD (P) H) and flavin adenine dinucleotide (FAD) are, respectively, the principal electron donors and acceptors of oxidative phosphorylation [12]. NAD (P) H has excitation maxima at 290 and 351 nm and emission maxima at 440 and 460 nm, while FAD has its excitation maxima at 450 nm and an emission maxima at 535 nm [13]. Measurement of fluorescence intensities for these excitation and emission profiles allows the comparison of the relative levels of FAD and NAD (P) H, the ratio of which (FAD/NAD (P) H, termed the redox ratio) is a non-invasive measure of metabolic activity which can indicate significant cellular characteristics including neoplastic status [14], quiescence and apoptosis [15], metastatic potential [16], and mesenchymal stem cell differentiation [17]. Beyond the assessment of optical redox ratio, measurement of autofluorescence has been used in the assessment of embryos [18], neurodegeneration [9], cartilage [19] and pain states [20].

In this project we apply a multispectral approach – exciting fluorophores with light sources from 340 to 660 nm and capturing cell images in selected spectral bands in the range from 440 to 715 nm – to collect image data from 34 spectral channels in total (channels are defined here as the emission signal received at a specific wave length due to excitation from a specific wavelength. This technology has been shown to be sensitive to changes in cell states and allow access to a greater range of complex information directly reflective of intracellular activity and composition than the conventional two-channel measurement of NAD (P) H and FAD [9, 10, 18]. Following multispectral imaging, cells were stained for DNA intensity and PCNA patterning to determine cell-cycle phases as a reference standard. The separate images were then matched on a cellular level to categorise the cells shown in the multispectral images to the different phases of the cell cycle. A model was then built to classify cell-cycle phase based on multispectral profile. Multispectral data was also unmixed to isolate the spectral signal of specific fluorophores and their variation across the cell cycle.

## Methods

This study aimed to define a multispectral signature for the non-invasive, non-destructive identification of cell cycle phase and investigate related changes in autofluorophores concentrations. A correlational microscopy approach was taken wherein cells were first imaged on a multispectral microscope, then fixed and stained immunohistochemically for markers of cell cycle phase. The same cells were then relocated and imaged again to provide a standard for cell cycle phase against which their multispectral characteristics could be referenced.

### Cell culture and preparation

Cells (HeLa, PANC1, and MIA-PaCa-2 purchased from Sigma-Aldrich and passaged < 10 times from thawing) were cultured in Dulbecco’s modified Eagle’s medium (DMEM) High glucose with L-glutamine (Gibco, Catalogue No: 11965–092) with 10% fetal calf serum (FCS; Gibco, Catalogue No. 26140–079) and Antibiotic-Antimycotic (Gibco, Catalogue No: 15240–062). Horse serum (Gibco, Catalogue No: 16050–122) at 2.5% was also included for the MIA-Pa-2 cells. Cell lines were chosen due to their widespread use as a model line (HeLa, a cervical cancer line) and to help elucidate the impact of tissue origin (PANC1 and MIA-PaCa-2; both pancreatic cancer cell lines).

Cultures were maintained in 5% CO_2_ at 37 °C. The detachment of cells from culture surfaces was carried out with TrypLE (Gibco, Catalogue No: 12604–021) after washing twice with Dulbecco’s PBS without calcium or magnesium (Sigma, Catalogue No: 14190–144). TrypLE was inactivated by adding twice its volume of growth media. For analysis 1 × 10^5^ cells were plated onto 35 mm ibiTreat glass bottomed dishes with 500 μm measurement grids (Ibidi, Catalogue No: 81166). Cells were given 24 h to attach and grow, then subjected to serum starvation (0.1% FCS) for 24 h to concentrate them in G1 phase. This method of synchronisation was utilised to minimise the potentially confounding impact that small molecule inhibitors could have if used to arrest cells at specific points in the cell cycle. Viability was confirmed by trypan blue staining (Gibco, Catalogu No: 15250061). They were then released by the addition of growth media.

Imaging was performed on parallel cultures over another 24 h to maximise the likelihood that data would be captured for sufficient numbers of cells for each phase of the cell cycle. Prior to imaging, cells were washed twice with Dulbecco’s PBS with calcium and magnesium (Sigma, Catalogue No: SLBS5504) to remove any traces of serum (which contains autofluorescent molecules and can confound assessment). Multispectral microscopy was then carried out with cells in Hank’s balanced salt solution (Gibco, Catalogue No: 14025–076).

### Multispectral microscopy

Multispectral microscopy was carried out using a Leica™ confocal system with a 40× oil objective lens (FLUOTAR340). Eighteen narrow band excitation wavelengths (excitation band ±5 nm) were selectively combined with four filter cubes (emission band: ±20 nm) for measuring emission to achieve 44 specific channels (Excitation (nm)/Emission (nm): 340/440, 368/440, 373/440, 378/440, 340/475, 368/475, 373/475, 378/475, 382/475, 388/475, 391/475, 394/475, 405/475, 413/475, 340/593, 368/593, 373/593, 378/593, 382/593, 388/593, 391/593, 394/593, 405/593, 413/593, 432/593, 441/593, 455/593, 460/593, 470/593, 491/593, 510/593, 413/715 (long-pass), 455/715 (long-pass), 660/715 (long-pass)).

Images of cells were manually segmented to define regions of interest based on differential interference contrast images taken at the same time as the multispectral images. To acquire the fluorescence spectral images, a CCD camera with high quantum efficiency (ORCA-Flash 4.0 LT) with a16bit A/D converter was used. We previously reported similar multispectral imaging using a custom-made multispectral microscopy system in the studies [9, 10, 21].

### Staining and confocal microscopy

Immunofluoresence staining for PCNA and DNA as the reference standard to indicate cell cycle phase was carried out according to a protocol adapted from Schonenberger et al. 2015 [6]. Cells were fixed in -20 °C methanol for 5 min then -20 °C acetone for 1 min followed by two washes in PBS. Fixation was carried out within 30 min of the initial multispectral image being taken to minimise the potential impact of cell cycle progression on results. Cells were then blocked in 1%BSA in PBS for 30 min at room temperature and exposed to the primary anti-PCNA antibody (rabbit polyclonal Abcam; Catalogue No: ab18197) for 2 h at room temperature at 1:500 in 10% normal goat serum (Gibco, catalogue No: 1620–064).

**Fig. 2:**
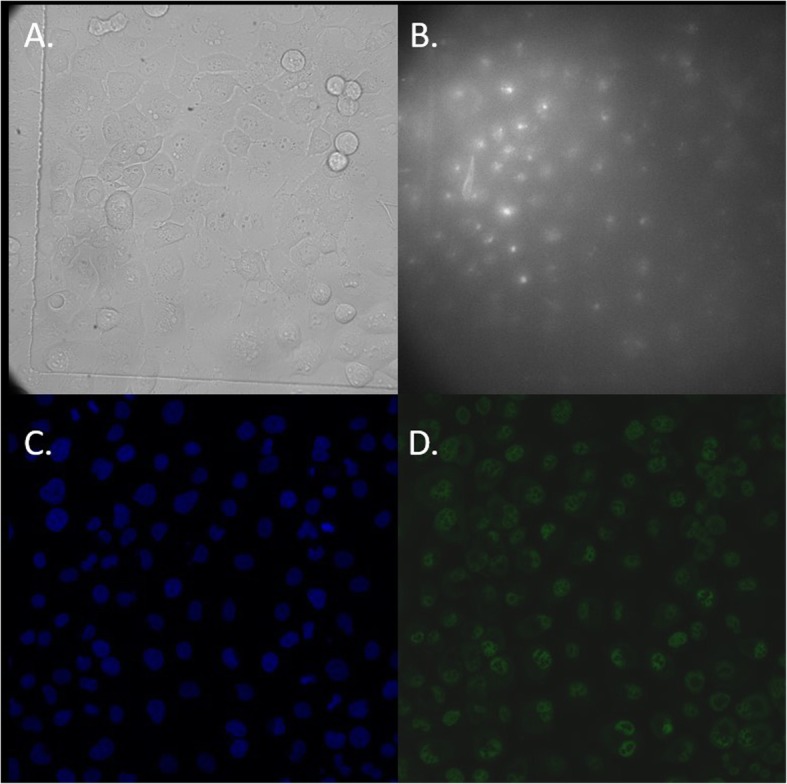
**a** Differential interference contrast from multispectral microscope of PANC1 stained cells at 13 h, **b**. Example multispectral channel 25 showing autofluorescence at excitation 432 nm emission 593 nm, **c**. Same field of view after staining with Dapi, taken using the confocal microscope, **d**. Same field of view after staining for PCNA, taken using confocal microscope. Regions of interest (cell area) were defined using (**a**), cell cycle phase was determined through measurement of fluorescence intensity in (**c**) and PCNA pattern in (**d**), then matched to data from multispectral channels including (**b**)

This was followed by a further two washes in PBS and exposure to the secondary antibody (goat polyclonal, Alexa Fluor 488, ThermoFisher, Catalogue No: A-11008) for 1 h at room temperature at 1:400 in 10% normal goat serum. Cells were washed twice with PBS and counterstained with DAPI for 5 min at room temperature before being imaged on a Leica SP2 confocal laser scanning microscope (Leica, Wetzlar, Germany) in PBS. Due to channel bleed, images for PCNA and DNA intensity were captured sequentially. For DNA intensity images the pinhole was opened entirely in order to capture total fluorescence. Cell cycle phase was determined through consideration of PCNA patterning (Fig. 1) as well as total DNA intensity. DNA intensity was measured using the graphics software ImageJ [22]. When phase had been determined images taken from the confocal microscope were compared to the differential interference contrast images from the multispectral microscope in order to match cell cycle phase to multispectral characteristics (Fig. 2). Trypan blue staining indicated that after serum starvation cell cultures retained viability (97% for HeLa, 97% for PANC1 and 99% for MIA-PaCa-2).

### Multispectral data analysis

The analysis of multispectral data in this study was carried out in three stages including image preparation, classification and autofluorescence un-mixing. Image preparation was undertaken as reported in our recent works [21, 23] to treat image artefacts, including Poisson’s noise, dead or saturated pixels, background fluorescence and illumination curvature. In subsequent analyses, the cells were segmented from the channel images to produce single cell images [24], and a variety of quantitative cellular image features were extracted. These features include mean channel intensity (e.g. cell image mean intensity of spectral channel with excitation 340 (nm) and emission 440 (nm)) and their associated statistical measures such as channel intensity ratio [10]. Further, features related to the histogram of the cell images such as pixels’ standard deviation and skewness in each spectral channel, which characterized the colour distribution of the cell images, were also considered [25]. In addition, to evaluate patterns within the cells – including repetitiveness or granularity – textural features of cell images such as entropy and homogeneity in each spectral channel were employed [26] (The mathematical definition of all features used in this study can be found in [10, 25, 26]). Such mathematically defined cellular features have been reported to be biologically significant in several studies [9, 10, 27] and they could capture various aspects of cell spectra and patterns in the cell images [9].

Next, depending on the classification target, indicative features which passed an ANOVA test (*P* < 0.005) and represented significant predictors were selected. The data points were then projected onto an optimal two-dimensional (2-D) space created by discriminative analysis [28]. This space maximizes between-group distance while minimizing within-group variance [28] and reducing the dimension of the selected feature vectors to two canonical variables which were equal to a linear combination of the selected cellular features [29]. Finally, a classifier was employed to predict the pre-defined cell labels [30, 31]. A linear classifier was used in this work due to its ability to deal with sparse data. This approach classified the cells based on a linear predictor function incorporating a set of weights obtained from the training process [32].

To provide the classifier with generalisability, a cross validation methodology was adopted [33] wherein data points were partitioned into 10 groups. Our linear classifier was developed based on 9 of these groups, and the tenth group was used for testing and the calculation of accuracy. Additionally, the receiver operating characteristic (ROC) graph was derived to determine the performance of this classifier as its discrimination threshold varied [21, 34].

### Unmixing of autofluorophores

Unmixing, in which the extracted spectral characteristics of the cells are compared to known characteristics of specific fluorophores (e.g. FAD, NAD (P) H at typical cellular concentrations) in order to calculate their abundance, was undertaken through a linear mixing model (LMM). This model assumes that the fluorescent signal of each pixel is a linear combination of a small number of endmember component spectra with respective weights corresponding to the concentration of the molecules responsible for these component spectra. These concentrations were expressed as abundance fractions [35–37]. The unsupervised unmixing algorithm, Robust Dependent Component Analysis (RoDECA) was used to identify the dominant native fluorophores and their corresponding abundance. RoDECA’s accuracy for isolating the spectral signal of specific fluorophores from the complex “noisy” cellular environment is established in Mahbub et al. 2017 [23]. As NADH and phosphorylated NADH have the same spectral properties they cannot be distinguished by this methodology and are collectively referred to as NAD (P) H.

## Results

### Differentiation of cell-cycle phases

**Fig. 3:**
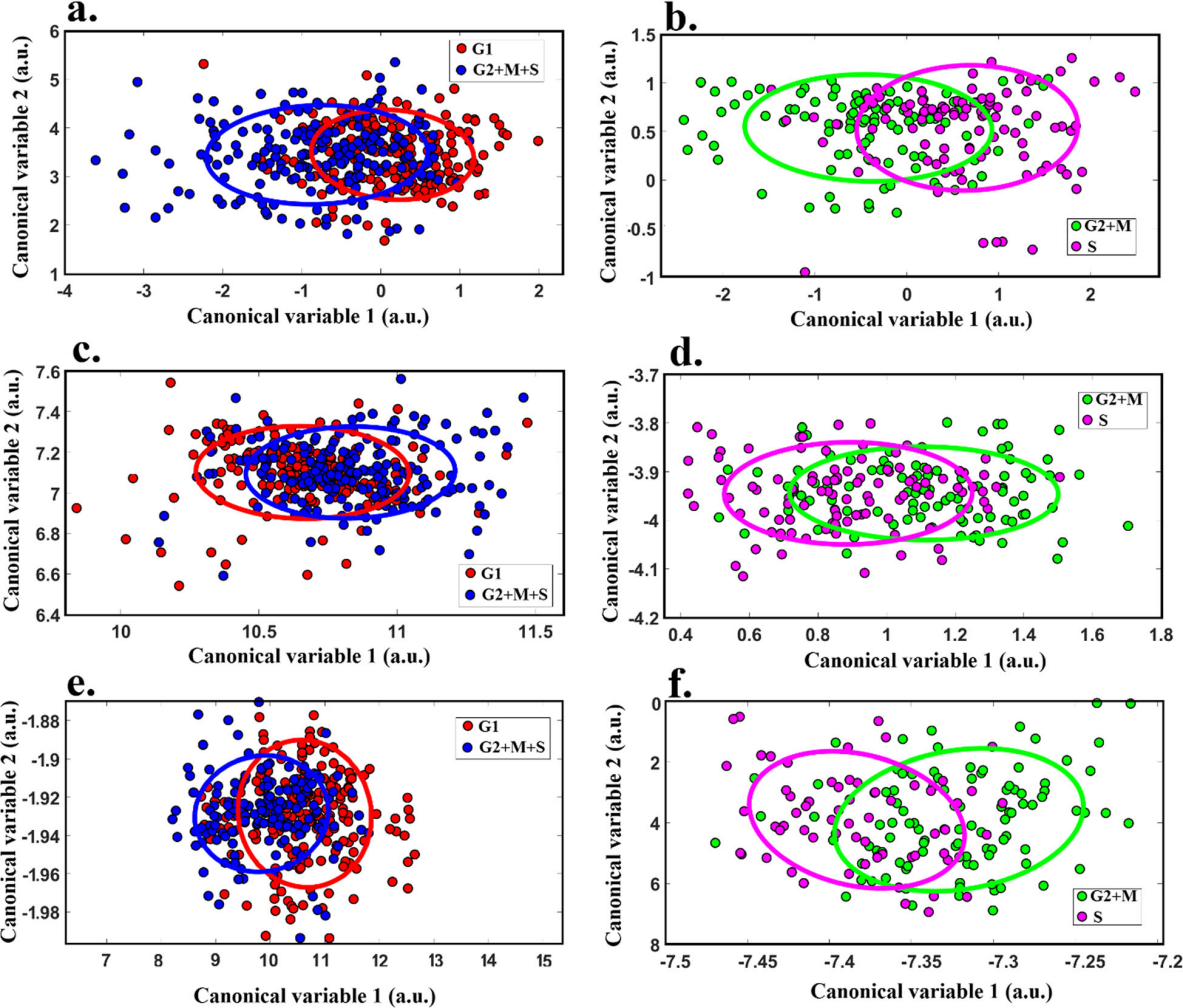
Cluster separation graphs and associated IoU values. Step 1 discrimination of G1 and S/G2 + M; **a**. hela cells (IoU = 31%), **c**. MIA-PaCa-2 (IoU = 55%), and **e**. PANC1 (39%). Step 2 discrimination of S and G2 + M; **b**. HeLa cells (IoU = 29%), **d**. MIA-PaCa-2 (45%), and **f**. PANC1 (28%) (*n* > 100)

Analyses were conducted at a single cell level where, based on PCNA patterning and DNA intensity, each cell was labelled as G1, S, or G2 + M (due to the comparative rarity of M-phase cells). A decision tree methodology was adopted to attain maximal separation between cell cycle phase groups based on differences in spectral features [38]. Accordingly, the classifications were done in two stages: first cells not in the process of replication were distinguished from those which were (i.e. G1 phase cells vs S, G2 and M phase cells) as they formed the most distinct initial cluster (area under the curve (AUC) > 75%), then S-phase and G2 + M-phase cells were differentiated from one another. In each step, the data points were reflected in the discrimination space created by corresponding canonical variables incorporating no more than 13 indicative features as shown in Fig. 3; these spaces were different for each pair of cell groups compared due to employing different features, as different features were indicative for different targets. To demonstrate the data distribution for each class, an ellipse has been defined for each cluster which illustrates the standard deviation of the data points. The overlap of the ellipses was quantified by their intersection over union (IoU) value, which is the ratio of the area of the two-ellipse intersection, divided by the area of their union. IoU values range from 0 to 100% for fully separated or overlapped ellipses, respectively.

**Fig. 4:**
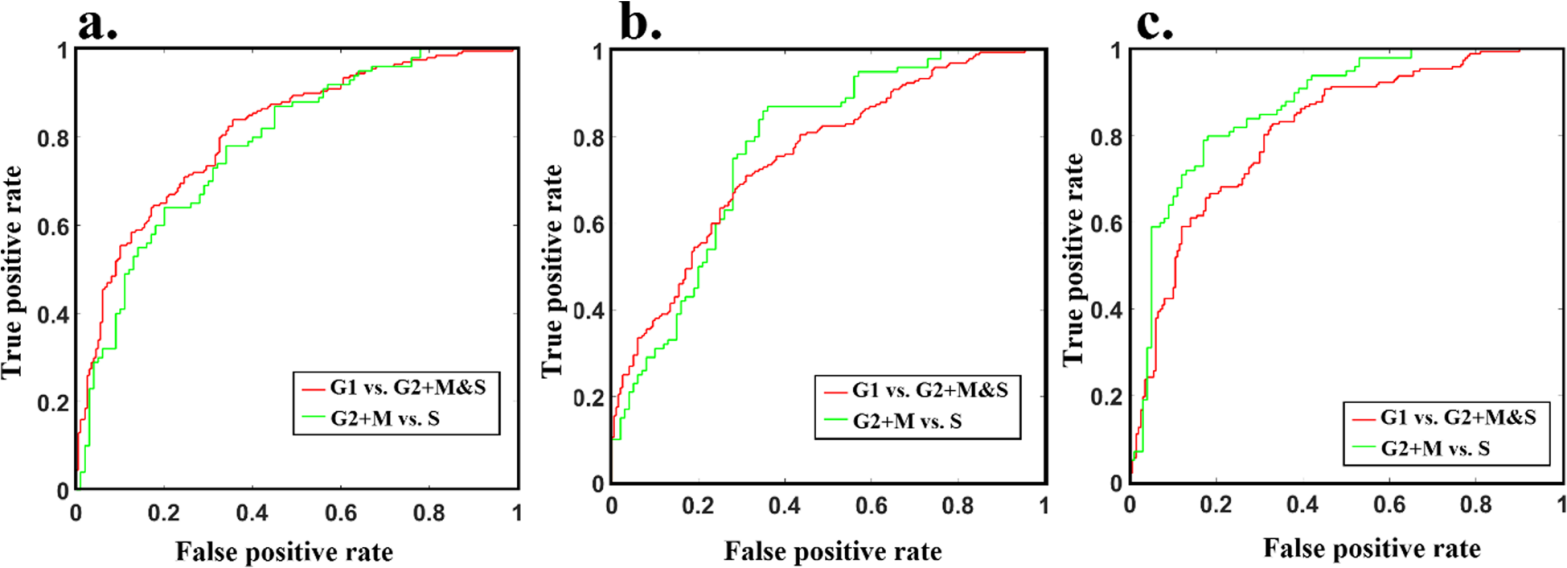
ROC curve for the accuracy of discrimination between cell cycle phases for **a**. hela, **b**.MIA-PaCa-2 and **c**. PANC1

IoU analyses showed that cell cycle phases can form separate clusters although a degree of overlap persists (Fig. 3). We then attempted to develop a linear classifier [30] that would determine a cell’s phase from its multispectral image. Figure 4 shows the receiver operating characteristic (ROC) graph which was derived to determine the performance of this classifier.

Overall, the classifiers for the different cell lines showed similar performance with average accuracies of 70.2 ± 2.2% and 72.6 ± 3.8 to classify G1 vs. S&G2 + M and S vs. G2 + M, respectively. The average AUCs for classifying G1 vs. S&G2 + M and G2 + M vs. S were 0.79 ± 0.28 and 0.80 ± 0.44 (Table 1). Sample size was >100 for all cell cycle phases across all cell lines. This approach achieved similar accuracy when an alternate approach to cell cycle manipulation (inhibitors instead of serum starvation) was applied (Additional file 1).

**Table 1:** Cell cycle classification performance

	HeLa		MIA-PaCa-2		PANC1 cell line	
	G1 vs. S&G2 + M	S vs G2 + M.	G1 vs. S& G2 + M	S vs G2 + M.	G1 vs. S& G2 + M	S vs.G2 + M
Accuracy	73.3%	71.0%	68.3%	69.0%	72.3%	78.0%
AUC	0.81	0.78	0.75	0.77	0.81	0.87

To provide comparison and context we applied a similar data analysis approach to classify cells by their tissue of origin (i.e. pancreatic (PANC1 and MIA-PaCa-2) or cervical (HeLa)) by spectral features as was applied to the cell cycle data. Initially, data points from the different cell origins were projected onto the discrimination space generated by canonical variables (specific for this analysis and different from all spaces illustrated in Fig. 4) and then a linear classifier was trained to predict cell origin. We emphasise that the feature subset used for cell origin classification was allowed to be different to the features selected for cell cycles as the subset of features which could differentiate cell cycles effectively would not be optimal for the discrimination of cell origin.

**Fig. 5:**
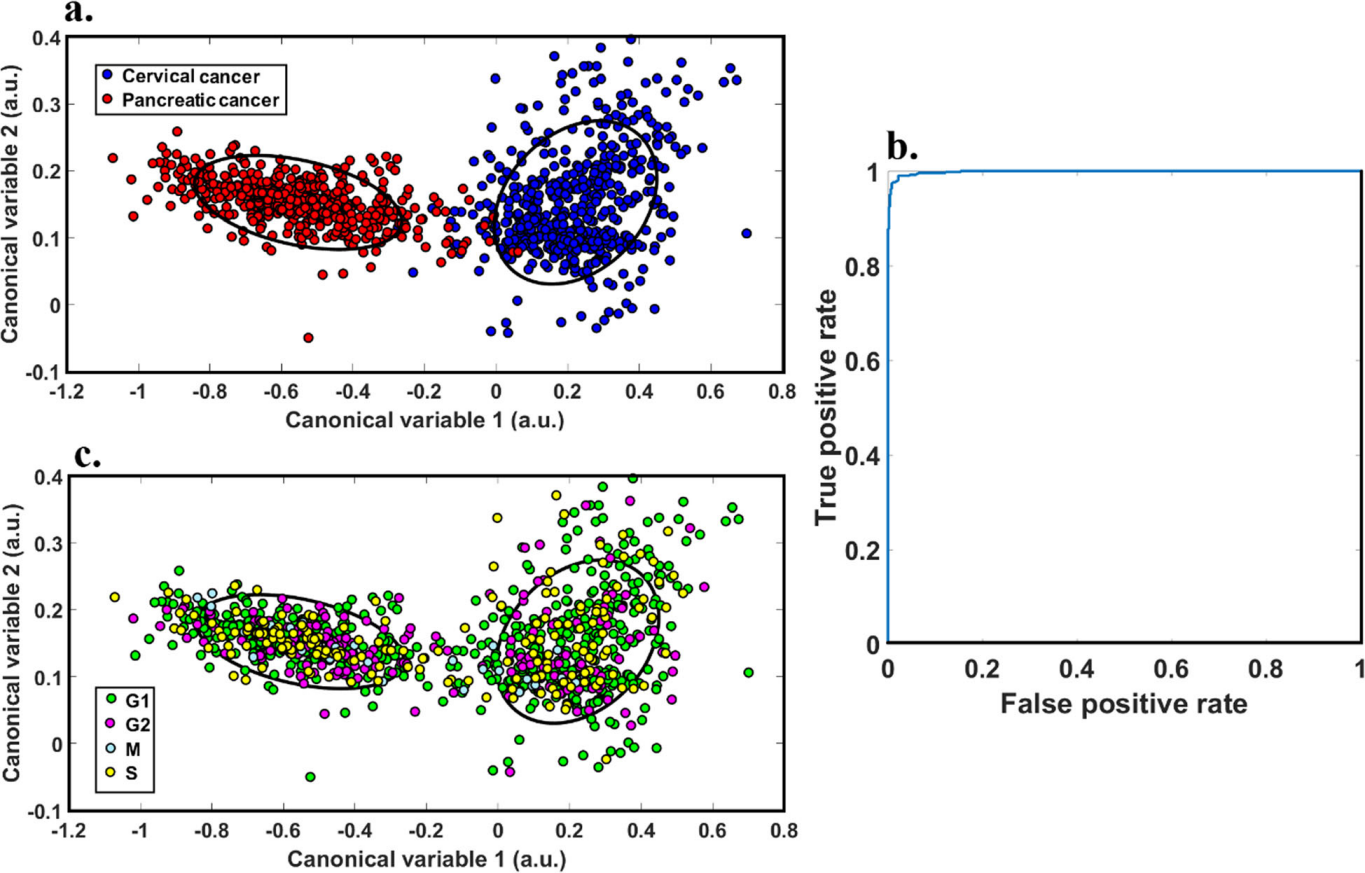
Discrimination of pancreatic cancer cells (MIA-PaCa-2 and PANC1) from cervical cancer cells (HeLa). **a** Cluster separation for pancreatic cancer cells (red) and cervical cancer (blue) with IoU = 0. **b**. ROC curve for the discrimination of pancreatic and cervical cancer cells. **c**. Cluster separation by cell origin with cell cycle phase indicated by colour

Of note, both pancreatic and cervical cancer formed two clearly separated clusters (IoU = 0 as shown in Fig. 5a), which demonstrates the strength of the spectral features used to discriminate between these cell origins. We then colour-coded each of the cells (Fig. 5c) based on the results of the cell cycle staining. When compared to the clusters formed by different phases of the cell cycle (Fig. 3) the strength of spectral features for cell origin differentiation is clearly much higher. The ROC obtained from linear classifiers trained for the different cell origins (Fig. 5) shows very high performance (AUC~ 1 & accuracy =97%). These observations imply that the spectral differences between different phases of the cell cycle, while observable (Fig. 4), are quite weak compared to spectral differences due to different cell origins. To show cell cycle with cell origin classification, the different cell phases were represented on the space discriminating cell types with different colours (Fig. 5c). This shows that the different cell cycle phases are randomly distributed between the clusters and do not form any meaningful subclusters. This is consistent with the separation of cell cycle phases occurring in a different space to that shown in Fig. 5a, c. This example shows that spectral differences due to different cell cycle phases may not interfere with cell origin classifications.

### Autofluorophore concentrations

**Fig. 6:**
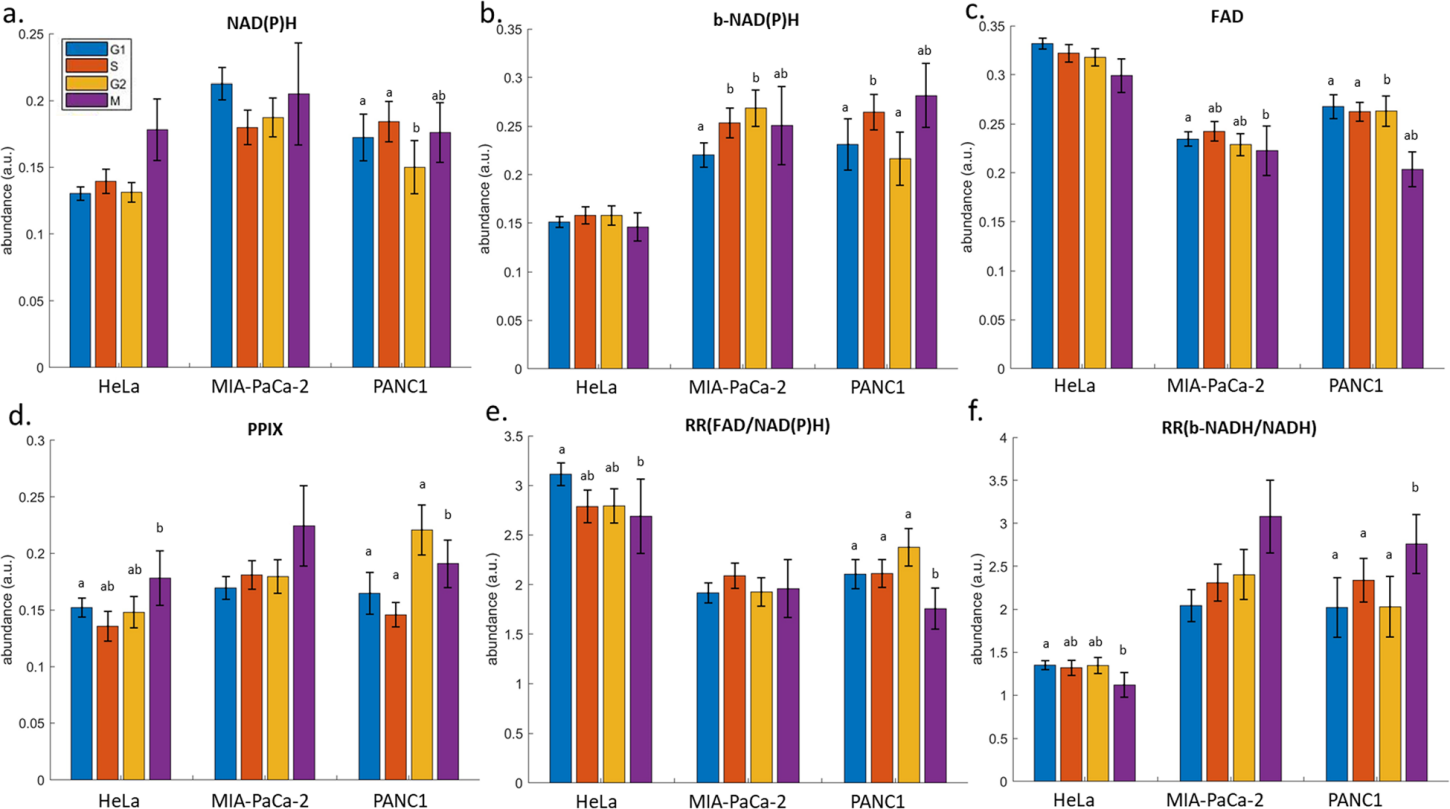
Autofluorophores across cell cycle phases for HeLa, MIA-PaCa-2 and PANC1 cells. **a**. NAD (P) H, **b**. protein bound NAD (P) H, **c**. FAD, **d**. PPIX, **e**. redox ratio (FAD/NAD (P) H), and **f**. protein bound NAD (P) H. Cell cycle phases are shown by different colours as indicated. Superscripts a and b differ at *p* < 0.05 according to a Mann-Whitney U test (two-tailed test, default). n ranged from 26 to 166, 23–196 and 34–50 for the phases within each cell line HeLa, MIA-PaCa-2 and PANC1 respectively

Autofluorophore unmixing of the multispectral data was successful in identifying NAD (P) H, protein bound NAD (P) H, FAD, and protoporphyrin IX (PPIX). The known cell cycle phase of cells – as determined through the DAPI and PCNA staining – was then used to calculate group averages. Although there were insufficient M-phase cells to model a multispectral signature for the identification of this phase separate to G2, there was sufficient power for group comparisons. The redox ratio (FADH/NAD (P) H) and ratio of bound NAD (P) H to NAD (P) H were also calculated (Fig. 6). Significant differences in autofluorophores were observed between cell cycle phases within cell lines, but no differences were consistently observed across all cell lines. Bound NAD (P) H was significantly elevated in S-phase cells compared to G1-phase cells in MIA-PaCa-2 and PANC1 cells, while M-phase cells had significantly higher PPIX than G1-phase cells in HeLa and PANC1 cells. Both the redox ratio and the ratio of bound NAD (P) H to free NAD (P) H were significantly higher in G1 compared to M-phase cells in HeLa and PANC1 cells.

## Discussion

This study investigated whether cell autofluorescence, measured by multispectral microscopy, could be used to discriminate between different phases of the cell cycle in neoplastic cells. We found that across different cancer cell lines accuracy ranged from 68.3% (MIA-PaCa-2) to 73.3% (HeLa) for distinguishing G1-phase cells from S and G2 + M-phase cells, and 69.0% (MIA-PaCa-2) to 78.0% (PANC1) for distinguishing S-phase cells from G2 + M phase cells (Fig. 3, Table 1). Unmixing of the multispectral data found autofluorophores which had significant differences between cell phases, including NADH, FAD, and PPIX (Fig. 6). Similarly, the redox ratio and the ratio of protein bound to free NADH were significantly affected by the cell cycle. The multispectral signature of cell cycle phase (Figs. 3 and 4) was a less accurate classifier compared to the signature for cell origin (Fig. 5), suggesting that strong differences between the spectral properties of cell populations or subpopulations are unlikely to be an artefact of differences in distribution across cell cycle phases.

Previous research on the spectral characterisation of the cell cycle examined the spectral properties of DNA stained cells that had been synchronised at different phases (G1, S, and G2/M with serum starvation, aphidicolin and nocodazole, respectively) [39]. They reported quantifiable spectral shifts in the DNA bound fluorophores between phases with consistent variations between lines and developed spectral signatures for the arrested cells, but did not investigate the accuracy of these signatures for the discrimination of cell cycle phases. Additionally, one study by Hsu et al. used multispectral Raman microscopy to investigate cytokinesis (the division of the cytoplasm between the two daughter cells at the end of M-phase) in human colon cancer cells [40]. They identified an autofluorescence signal – proposed to be from a lipid – that was concentrated in the vicinity of the cytokinesis cleavage furrow, which could potentially be used for the label-free identification of cells at the end of M-phase.

We observed that, for all cancer cell lines examined, the multispectral signatures of S and G2 + M-phase cells tended to cluster together, apart from the signature of G1-phase cells. As the cell transitions through the G1-S restriction point growth-dependent cyclin dependent kinase (CDK) – responsible for the promotion of DNA replication – initiates a positive feedback loop which increases CDK activity and commits the cell to division, independent of further environmental signals, through the induction of genome-wide transcriptional changes [41]. Although other waves of transcription occur during the G2-M and M-G1 transitions, there is no major effect at the S-G2 transition [41–43]. This lack of a change in transcriptional activity, despite the completion of DNA-synthesis and the initiation of G2-phase growth, could be why the multispectral characteristics of G1-phase cells formed a distinct cluster relative to S and G2 + M-phase cells. M-phase cells were included with G2 phase cells in our data-sets, but due to their low numbers are not likely to have impacted this observation. Mutations in regulatory proteins involved in the G1-S transition are found with high frequency in cancer cells [44–46] as overcoming this checkpoint’s control of growth is a key step in the development of cancer. If the clustering we have observed does relate to changes experienced by cells on passing through this checkpoint further investigation of its nature between neoplastic cell lines, and in comparison to non-neoplastic cells, may open a novel route for research into oncogenesis.

Cell cycle control and metabolism have a tight, bidirectional relationship, with the ability of the cell to commit to growth depending on the availability of metabolites, and the molecular mechanisms of the cell-cycle being linked to the regulation of metabolic networks [47]. In the present study the redox ratio did not differ between G1 and S or G2 phase for any of the lines (Fig. 6e) and it was increased in G1 compared to M phase cells for HeLa and PANC1. Similarly, in PANC1 cells the redox ratio was significantly higher in S and G2 phase cells compared to M-phase cells. A previous work by Datta et al. 2018 [11] made the same finding for G1 compared to M phase cells in the HeLa cell line (although they used the inverse formula for redox ratio (NADH/FAD) to the definition we applied and consequently report an increase in redox ratio for M phase). They also reported the same difference between G2 and M phase cells that we observed in the PANC1 line.

Additionally, Datta et al. 2018 [11] assessed the effect of the cell cycle on the relative proportion of NADH existing in a free or protein bound state in HeLa cells. They used fluorescence-lifetime imaging which utilises the increased lifetime of protein bound NADH to distinguish it from unbound NADH and found a decreased mean lifetime of NADH – corresponding to a relative increase in free NADH – during M-phase. Our results did not show a significant difference in free or bound NAD (P) H in HeLa cells during any phase (Fig. 6f). However, the ratio of bound versus free was significantly decreased in M phase compared to G1 suggesting increased free NAD (P) H relative to bound. Differences were non-significant for MIA-PaCa-2 cells. In contrast M-phase PANC1 cells exhibited significantly elevated ratios of bound NAD (P) H compared to free, relative to all other cell phases.

Unmixing was also able to successfully identify the signal for protophorin IX (PPIX; Fig. 6d). Previous work in bladder cancer cells showed a significant decrease in PPIX in G1 cells compared to S or G2 and M phase cells [48]. We observed no significant differences in MIA-PaCa-2 cells, however PPIX was elevated in M-phase cells compared to G1 in HeLa and PANC1. Additionally, in PANC1 PPIX was significantly increased in G2 phase compared to M, and M phase compared to S. Differences in changes in PPIX concentration between phases of the cell cycle in different cancer cell lines could be important as it acts as a photosensitiser in photodynamic therapy [49].

The final models were not able to achieve full discrimination between cell cycle phases in the cancer cell lines examined. This could potentially be improved through the use of a higher resolution camera to reduce image noise or the excitation of autofluorescence across a larger number of channels covering a broader range of wavelengths. However, high genomic and transcriptomic heterogeneity exists within neoplastic cell lines [50, 51] and appears to be exacerbated with expansion [51]. As such, between-cell variation independent of cell cycle phase is likely to have confounded discrimination. If this was the case the use of multispectral microscopy to assess cell cycle phase would be expected to be more accurate in more recently cultured cancer cell lines, tumour biopsies and, if attempted, in vivo tumour imaging – although in the latter case increased biological variability and the added complexity of 3D structures will present further challenges and require significant technological advances in terms of automated image smoothing and the algorithmic discrimination of regions of interest (i.e. discrimination of connective tissue from organ). If realised, however, this advance would be of considerable therapeutic value for cancer diagnostics and characterisation.

The developed models, although validated in separate testing data-sets to those used to train them, can only be considered to apply to the cell line they were created for, and we found them to not be generalizable. These specific models achieved moderate success, but if a more generalizable model was attempted – through the use of multiple cell lines in its training – the loss of ‘custom tailoring’ of the model to cell line makes it unlikely that a useful level of discrimination could be achieved under the same conditions. Finally, although a high enough number of cells were imaged and correlated for the creation of both training and testing data-sets, too few M-phase cells were captured for them to be considered separately to G2-phase cells. This is not a major disadvantage for this system as it still matches the level of discrimination of the most frequently used methods of cell cycle phase investigation, with the advantages of being able to be carried out in live, plated cells without use of reporters or stains. However, further work with larger data-sets could overcome this problem, and the separation of M-phase from G2-phase could improve overall discrimination, as if they are multispectrally distinct – and the G2-M transition transcription wave [41] suggests that this should be the case – their combination could be complicating discrimination.

## Conclusions

This study has shown that the multispectral measurement of cell endogenous autofluorescence is able to discriminate live cancer cells’ stages in the cell cycle without detachment, labelling or transformation – representing considerable advantages over traditional approaches including FLOW cytometry and fluorescence microscopy. This finding represents a first potential step towards minimally invasive assessment of cell division – a useful characteristic for treatment planning. Signatures of cell autofluorophores were successfully unmixed and showed significant differences between cell-cycle phases, however these differences were not consistent between cell lines and suggest that the creation of a generalisable, reliable signature of cell cycle phases that can be applied across cancer cell lines may be complicated. As well as having practical applications for cancer cell research, these results provide novel information on the mechanisms and characteristics of the phases of the cell cycle.

## Supplementary information

**Additional file 1.** Cell cycle differentiation in cells exposed to cell cycle inhibitors.

## Data Availability

The datasets used during the current study are available from the corresponding author on reasonable request.

## Abbreviations

AUC: Area under the curve
CDK: Cyclin dependant kinase
DMEM: Dulbecco’s modified Eagle’s medium
FAD: Flavin adenine dinucleotide
FCS: Fetal calf serum
IoU: Intersection over union
LMM: Linear mixed modelling
NAD (P) H: Nicotinamide adenine dinucleotide
PCNA: Proliferating cell nuclear antigen
PPIX: Protoporphyrin
ROC: Receiver operating characteristic
RoDECA: Robust Dependent Component Analysis
