# Supplementary material for: Non-destructive, label free identification of cell cycle phase in cancer cells by multispectral microscopy of autofluorescence
Source: BMC Cancer. 2019 Dec 21;19:1242. doi: 10.1186/s12885-019-6463-x (PMC6925881; doi:10.1186/s12885-019-6463-x)
Supplement: Supplementary file 1 — Additional file 1. Cell cycle differentiation in cells exposed to cell cycle inhibitors. [file 12885_2019_6463_MOESM1_ESM.docx]

**Supplementary materials**

Supplementary material 1: ***Analysis pipeline***

**Imaging:** Multispectral with immunofluorescence for correlation

**Image preparation:** Address image artefacts, including Poisson’s noise, dead or saturated pixels, background fluorescence and illumination curvature

**Segmentation:** Manual definition of regions of interest and correlation to fluorescent imaged for cell cycle phase classification.

**Feature extraction:** Features to include; mean channel intensity, channel intensity ratio, color distribution, and textural features.

**Discriminative analysis:** Project on an optimised 2-D space which maximizes between-group distance and minimises within-group variance to reduce the dimension of selected feature vectors to two canonical variables which are equal to a linear combination of the selected features.

**Classification:** For sparse data use a linear classifier to classify cells based on a linear predictor function incorporating a set of weights obtained in a training process. Apply a cross-validation methodology wherein data points are partitioned into 10 groups. Develop the classifier using 9 of these groups and test on the tenth.

Supplementary material 2: ***Cell cycle inhibitor***


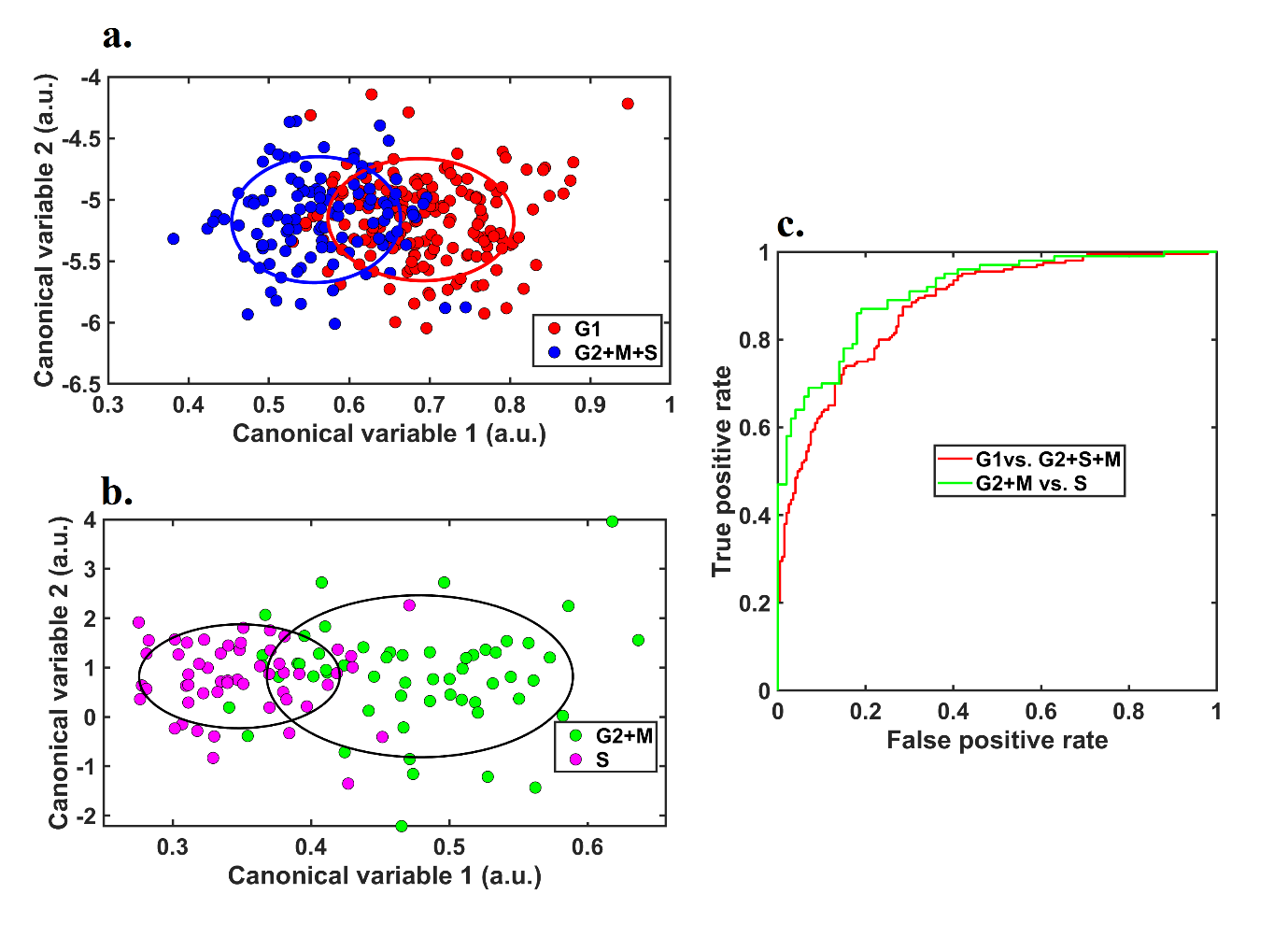


Supplementary figure 1: Classifying cell cycle phase in cells treated with cell cycle inhibitors; a) Cluster separation graph for G1 from S or G2+M, b) Cluster separation graph fro S from G2+M, c) ROC curve for the accuracy of discrimination between cell cycle phases

Supplementary table 1: Cell cycle classification performance

|  | **Cell cycle inhibitor PANC-1** | |
| --- | --- | --- |
|  | *G1 vs. S&G2+M* | *S vs G2+M.* |
| ***Accuracy*** | *76%* | *79%* |
| ***AUC*** | *0.87* | *0.90* |
| ***IoU*** | *17%* | *9%* |

PANC-1 cells were treated for 24h hours with the cell cycle inhibitors aphidicolin (5µM), camptothecin (1µM), and etoposide (1µM) separately. Cells were then assessed using multispectral microscopy and immunofluorescence as described in the methodology. Similar accuracy was obtained for the resultant model (supplementary table 1) as when cells were synchoronised using serum starvation then released.

Spectral microscopy was carried out on an Olympus IX83 microscope with a NuVu electron multiplying charge coupling device (EMCCD, hnu1024) camera, a 40× oil objective lens (UAPON340, Olympus).
